# Factors Associated with Sugar-Sweetened Beverage Intake Among Young Children – United States, 2021

**DOI:** 10.5888/pcd21.230354

**Published:** 2024-03-21

**Authors:** Mary Ellen Grap, Heather C. Hamner, Carrie Dooyema, Adi Noiman, Sohyun Park

**Affiliations:** 1 Division of Nutrition, Physical Activity, and Obesity, National Center for Chronic Disease Prevention and Health Promotion, Centers for Disease Control and Prevention, Atlanta, Georgia; 2 Oak Ridge Institute for Science and Education, Oak Ridge, Tennessee; 3 Epidemic Intelligence Service, Centers for Disease Control and Prevention, Atlanta, Georgia

## Abstract

**Introduction:**

Because limited data exist about factors related to sugar-sweetened beverage (SSB) intake among younger children, we investigated factors associated with SSB intake among US children aged 1 to 5 years.

**Methods:**

We examined SSB intake (0, 1–3, or ≥4 times/week) by using data from the 2021 National Survey of Children’s Health. We performed a multinomial logistic regression to calculate adjusted odds ratios (aORs) for select sociodemographic and household factors associated with moderate (1–3 times/week) and high (≥4 times/week) SSB intake.

**Results:**

Overall, 36% of children consumed SSBs 1 to 3 times/week and 21% consumed 4 or more times/week. Both moderate and high SSB intake were associated with child’s age, child’s race and ethnicity, highest caregiver education level, household income, primary household language, and frequency of family meals. For example, children who lived in households with caregiver education level of high school graduate or less were significantly more likely to have moderate (aOR, 2.06) and high (aOR, 2.81) SSB intake than those who lived in households with caregiver education level of college degree or higher. High SSB intake was also associated with marginal household food sufficiency, nonmetropolitan statistical area status, and receipt of government food benefits.

**Conclusion:**

Several sociodemographic and household factors were significantly associated with SSB intake among children aged 1 to 5 years. Public health initiatives designed to address SSB intake among young children in various settings including pediatric health care, early care and education, and the child’s home could consider key associated factors.

**Summary**

**What is already known on this topic?**

Optimal nutrition is key for child health and development. Added sugars, of which sugar-sweetened beverages (SSBs) are leading sources, are associated with adverse health outcomes. Sociodemographic characteristics have been associated with SSB intake for adolescent and adult populations.

**What is added by this report?**

This study found that both moderate (1–3 times/week) and high (≥4 times/week) SSB intake was associated with a child’s age, race and ethnicity, and caregiver’s education level; household income; primary household language; and frequency of family meals.

**What are the implications for public health practice?**

Public health initiatives aimed at reducing SSB intake could consider multiple sociodemographic and household factors associated with SSB intake among this population.

## Introduction

The first 5 years of life are a critical period for children’s growth, health, and cognitive development (1). Good nutrition is essential during this period. The US Dietary Guidelines for Americans (DGAs) provide dietary recommendations throughout the lifespan. The 2020–2025 DGAs recommend that children aged less than 2 years avoid foods or beverages with added sugars and that children aged 2 years or older limit intake of added sugars to less than 10% of total daily calories (1). National data suggest that US children are exceeding recommendations for added sugars (2,3).

Sugar-sweetened beverages (SSBs) are primary sources of added sugars in the diet of Americans (4). SSBs include any liquids that are sweetened with added sugars, such as soda, energy drinks, sports drinks, teas, and fruit drinks (1). Increased consumption of SSBs is associated with adverse health outcomes in children and adults, including increased risk for obesity, type 2 diabetes, dental caries, cardiovascular disease, and asthma (5–7).

A previous study found that 57% of children aged 1 to 5 years consumed 1 or more SSBs during the previous week in 2021 (8). Weekly SSB consumption varied by geography, age, race and ethnicity, and household food sufficiency (8). Previous studies have explored factors associated with SSB intake among youth and adult populations. Studies focused on youth populations (aged 2–19 years) have assessed the association of SSB intake with factors including maternal dietary habits (9–11), taste preference (9,10), screen time (9,10,12), proximity to a fast-food restaurant (10–12), and availability of SSBs in school settings (13). Differences in SSB intake were reported by race and ethnicity and socioeconomic status in youth and adult populations (5,8,10–13). However, there is very limited information on sociodemographic and household factors associated with SSB intake among younger children that comes from nationally representative data, especially for those aged less than 2 years. Therefore, we examined factors associated with SSB intake in a nationally representative sample of US children aged 1 to 5 years.

## Methods

### Sample and survey administration

The National Survey of Children’s Health (NSCH) is an annual, nationally representative survey of noninstitutionalized children aged 0 to 17 years that provides national and state-specific estimates (14). It is sponsored by the Maternal and Child Health Bureau of the Health Resources and Services Administration and administered by the US Census Bureau. NSCH provides data on physical and emotional health of children aged 0 to 17 years. Survey topics include characteristics of the child, family, and neighborhood. Households complete a screening questionnaire to determine eligibility. Among those eligible, an age-specific topical questionnaire (0–5, 6–11, or 12–17 years) is completed by an adult caregiver online, via email, or over the phone. Adult caregivers answer questions about the health and well-being of 1 randomly selected child in the household (14). Data for the 2021 survey were collected from June 25, 2021, to January 14, 2022. Overall, the weighted response rate was 40.3% in 2021. The weighted topical completion rate was 32.3% (14).

We used 2021 survey data and limited analyses to children aged 1 to 5 years (n = 18,830). Age in years was defined as the following ranges in months: 1 year of age was defined as 11 to 23 months, 2 years as 24 to 35 months, 3 years as 36 to 47 months, 4 years as 48 to 59 months, and 5 years as 60 to 71 months. Those who had a missing response to the SSB question (n = 279) were excluded, leaving a final analytic sample of 18,551 children.

### Outcome variable

In 2021, SSB intake, the outcome of interest, was asked for the first time and only among children aged 1 to 5 years of age. Adult caregivers were asked “During the past week, how many times did this child drink sugary drinks such as soda, fruit drinks, sports drinks, or sweet tea? Do not include 100% fruit juice.” (15). Response options were discrete, and the respondent could only select a single choice from the following options: this child did not drink sugary drinks; this child consumed sugary drinks 1 to 3 times during the past week; 4 to 6 times during the past week; 1 time/day; 2 times/day; or 3 or more times/day (15). Response options were recategorized as 0 times/week, 1 to 3 times/week, or 4 or more times/week. This was done to compare no intake, moderate (defined as 1–3 times/week), and high (≥4 times/week) intake levels. Cut points for SSB intake were chosen based on the data distribution.

### Independent variables

Demographic variables included child’s age (1, 2, 3, 4, 5 years), maternal age (≤24, 25–29, 30–34, ≥35 years), child’s sex (male, female), and the selected child’s race and ethnicity as reported by the parent or caregiver (non-Hispanic Asian, non-Hispanic Black, Hispanic, non-Hispanic other/multiracial [included American Indian/Alaska Native, Native Hawaiian and other Pacific Islander, and multiple race], non-Hispanic White). Other variables included were the highest level of education among reported adult caregivers in the household, referred to hereafter as highest household caregiver education (less than high school graduate or high school graduate/General Educational Development [GED] certificate, some college or technical school, college degree or higher), household income (<130% federal poverty guidelines [FPG], 130% to <350% FPG, ≥350% FPG), primary household language (English, Spanish, other), food situation in the past 12 months (food sufficient: could always afford to eat good nutritious meals; marginal food sufficiency: could always afford enough to eat but not always nutritious meals; low food sufficiency: often or sometimes could not afford enough to eat), frequency of family eating meals together during the past week (0–3 days/week, 4–6 days/week, every day), metropolitan statistical area status (yes, no; yes was defined as having at least 1 urbanized area of ≥50,000 population and surrounding counties having strong economic and social ties to the core area [16]), and any government food assistance benefits (Supplemental Nutrition Assistance Program [SNAP]; the Special Supplemental Nutrition Program for Women, Infants, and Children [WIC]; free or reduced-cost breakfast or lunch benefits) for anyone in the household in the past 12 months (yes, no).

### Statistical analysis

We used descriptive statistics to estimate the proportion of children consuming SSBs overall and by each characteristic. Differences in SSB intake were identified by using χ^2^ tests; *P* < .05 was considered significant. Multinomial logistic regression modeling was done to estimate adjusted odds ratios and 95% CIs for factors associated with SSB intake 1 to 3 times/week and 4 or more times/week, using non-SSB consumers (0 times/week) as a reference group. All sociodemographic characteristics and other factors were included in one model; 16,209 children who had complete data for all variables were included in the model. NSCH uses weighting to account for nonresponse and for population and demographic controls (17). SAS-callable SUDAAN version 11 (RTI International) was used to account for the complex survey design.

This study was a secondary analysis with publicly available deidentified data, and therefore was not considered human subjects research by the Centers for Disease Control and Prevention and did not require institutional review board review.

## Results

Among our unweighted analytic sample of US children aged 1 to 5 years, 13% were 1 year of age, 23% were 2 years of age, 21% were 3 years of age, 22% were 4 years of age, and 22% were 5 years of age. Among children aged 1 to 5 years, 43% did not consume any SSBs in the previous week, 36% consumed SSBs 1 to 3 times/week (moderate intake), and 21% consumed SSBs 4 or more times/week (high intake) (Table 1). SSB intake was significantly different for all covariates except for sex. For example, the distribution of children’s age was significantly different across categories of SSB intake. Among 1 year old children, 69% consumed SSBs 0 times/week; 21%, 1 to 3 times/week; and 10%, 4 or more times/week. Among 5-year-old children, 27% consumed SSBs 0 times/week; 47%, 1 to 3 times/week; and 26%, 4 or more times/week.

**Table 1:** Characteristics of Children Aged 1 to 5 Years and Their Associations With Sugar-Sweetened Beverage Intake During the Past 7 Days, National Survey of Children’s Health, 2021

Characteristics	All, n (%)^b^	Sugar-sweetened beverage intake^a^			
		0 times/week	1–3 times/week	≥4 times/week	*P* value^c^
		%^b^ (SE)	%^b^ (SE)	%^b^ (SE)	
**Total (N = 18,551)^d^**		42.6 (0.9)	36.4 (0.9)	21.0 (0.8)	NA
**Child’s age**					
1 year (12–23 months)	2,456 (18.8)	69.1 (2.3)	20.9 (2.1)	10.0 (1.3)	<.001
2 years (24–35 months)	4,261 (19.9)	48.4 (1.8)	35.1 (1.8)	16.5 (1.4)	
3 years (36–47 months)	3,836 (20.4)	38.4 (1.8)	37.7 (2.0)	23.9 (1.8)	
4 years (48–59 months)	4,013 (21.3)	32.0 (1.8)	40.3 (2.0)	27.7 (2.2)	
5 years (60–71 months)	3,985 (19.6)	27.3 (1.7)	47.1 (1.9)	25.6 (1.8)	
**Child’s sex**					
Boys	9,636 (51.2)	42.5 (1.2)	36.0 (1.2)	21.5 (1.1)	.78
Girls	8,915 (48.8)	42.8 (1.3)	36.8 (1.3)	20.4 (1.2)	
**Child’s race and ethnicity**					
Asian, non-Hispanic	1,056 (4.9)	43.9 (3.5)	39.5 (3.8)	16.6 (2.7)	<.001
Black, non-Hispanic	1,075 (13.0)	28.2 (2.7)	39.6 (2.8)	32.2 (2.6)	
Hispanic	2,438 (25.4)	32.4 (2.2)	38.8 (2.4)	28.8 (2.3)	
Other/multiracial, non-Hispanic^e^	1,580 (6.9)	52.4 (2.6)	33.3 (2.3)	14.4 (1.9)	
White, non-Hispanic	12,402 (49.7)	50.2 (0.9)	34.5 (0.9)	15.3 (0.7)	
**Maternal age, y**					
≤24	2,213 (16.3)	32.7 (2.4)	37.9 (2.7)	29.4 (2.7)	<.001
25–29	1,926 (10.9)	33.5 (2.4)	41.6 (2.7)	24.9 (2.3)	
30–34	4,762 (22.3)	46.0 (1.5)	35.4 (1.5)	18.6 (1.3)	
≥35	9,650 (50.5)	46.3 (1.3)	35.2 (1.2)	18.4 (1.1)	
**Highest level of education among adults in the household**					
High school graduate/GED or less	2,524 (27.3)	24.5 (1.7)	42.4 (2.2)	33.1 (2.2)	<.001
Some college or technical school	3,553 (18.0)	33.1 (1.7)	39.7 (1.8)	27.2 (1.9)	
College degree or higher	12,474 (54.7)	54.9 (1.0)	32.3 (1.0)	12.8 (0.7)	
**Household income^f^**					
<130% FPG	2,933 (25.3)	27.0 (1.9)	40.1 (2.2)	32.9 (2.2)	<.001
130% to <350% FPG	6,403 (36.3)	39.0 (1.4)	38.8 (1.5)	22.2 (1.3)	
≥350% FPG	9,215 (38.3)	56.4 (1.2)	31.7 (1.1)	11.8 (0.9)	
**Primary household language (n = 18,481)**					
English	16,962 (84.2)	45.2 (0.9)	35.7 (0.9)	19.1 (0.7)	<.001
Spanish	686 (9.9)	20.2 (2.6)	43.4 (4.2)	36.4 (4.3)	
Other	833 (5.8)	44.7 (4.5)	34.7 (4.5)	20.6 (4.4)	
**Household food situation during the past 12 months (n = 18,166)**					
Food sufficient: could always afford to eat good nutritious meals	14,566 (75.3)	46.8 (1.0)	36.1 (1.0)	17.1 (0.9)	<.001
Marginal food sufficiency: could always afford enough to eat but not always nutritious meals	3,245 (21.5)	30.8 (1.9)	37.1 (2.1)	32.1 (2.1)	
Low food sufficiency: often or sometimes could not afford enough to eat	355 (3.2)	28.5 (4.5)	35.3 (4.9)	36.2 (5.2)	
**Frequency of family eating meals together during the past week (n = 18,275)**					
0–3 days/week	2,759 (16.9)	35.8 (2.1)	39.4 (2.3)	24.8 (1.9)	.005
4–6 days/week	4,424 (22.5)	43.9 (1.8)	37.4 (1.7)	18.7 (1.8)	
Every day	11,092 (60.6)	44.3 (1.1)	34.9 (1.1)	20.8 (1.1)	
**Metropolitan statistical area status (n = 16,631)**					
Metropolitan statistical area	13,644 (88.2)	43.5 (1.0)	36.0 (1.0)	20.5 (0.9)	<.001
Nonmetropolitan statistical area	2,987 (11.8)	34.0 (1.8)	39.7 (2.1)	26.4 (1.8)	
**Government food assistance benefits for anyone in the household in the past 12 months^g^ (n = 18,220)**					
Yes	5,141 (40.6)	29.7 (1.4)	40.0 (1.6)	30.3 (1.6)	<.001
No	13,079 (59.4)	51.7 (1.0)	33.8 (1.0)	14.5 (0.7)	

Abbreviations: FPG, federal poverty guidelines; GED, General Educational Development certificate; NA, not applicable.

a “During the past week, how many times did this child drink sugary drinks such as soda, fruit drinks, sports drinks, or sweet tea? Do not include 100% fruit juice.”

b Percentages based on weighted sample size. Weighted percentage may not add up to 100 because of rounding.

c χ^2^ tests were used for each variable to examine differences across categories and *P* < .05 was considered significant.

d Unweighted sample size.

e Included American Indian/Alaska Native, Native Hawaiian and other Pacific Islander, and multiple race.

f This variable represents household income as a percentage of the federal threshold by family composition. This variable was multiply imputed according to National Survey of Children’s Health guidelines to account for missing values. We recategorized the cutoffs to reflect the cut point for participation in the Supplemental Nutrition Assistance Program (SNAP) and provide relatively equal sample sizes for each income group.

g This variable reflects participation in SNAP or Special Supplemental Nutrition Program for Women, Infants, and Children (WIC), or free or reduced cost breakfast or lunch benefits in the previous 12 months.

After adjusting for other covariates, several factors were significant in the multinomial logistic regression model (Table 2). Higher odds of moderate SSB intake were significantly associated with children being older (vs 1 year of age), non-Hispanic Asian or non-Hispanic Black (vs non-Hispanic White), maternal age of 25 to 29 years (vs ≥35 years), highest caregiver education of high school graduate/GED or less or some college or technical school (vs college degree or higher), household income of 130% to less than 350% (vs ≥350%) of FPG, primary household language of Spanish (vs English), and fewer family meals per week (vs every day) (Table 2). The odds of moderate SSB intake were higher for children in a household with Spanish as the primary language compared with English as the primary language (adjusted odds ratio [aOR], 1.84; 95% CI, 1.14–2.99) and for children in households reporting frequency of family meals 0 to 3 days/week (aOR, 1.43; 95% CI, 1.12–1.83) compared with family meals every day.

**Table 2:** Factors Associated With Sugar-Sweetened Beverage Intake During the Past 7 Days Among Children Aged 1 to 5 Years Based on Multinomial Logistic Regression Analysis, National Survey of Children’s Health, 2021 (n = 16,209)

Characteristics	Sugar-sweetened beverage intake^a^, adjusted odds ratio (95% CI)^b^	
	Moderate intake (1–3 times/week)	High intake (≥4 times/week)
**Child’s age**		
1 year (12–23 months)	1 [Reference]	1 [Reference]
2 years (24–35 months)	2.78 (2.07–3.72)	3.22 (2.21–4.71)
3 years (36–47 months)	3.47 (2.56–4.70)	5.40 (3.67–7.96)
4 years (48–59 months)	4.81 (3.56–6.49)	7.97 (5.37–11.82)
5 years (60–71 months)	6.38 (4.63–8.80)	8.68 (5.75–13.11)
**Child’s sex**		
Boys	1 [Reference]	1 [Reference]
Girls	1.00 (0.85–1.18)	0.93 (0.75–1.15)
**Child’s race and ethnicity**		
Asian, non-Hispanic	1.65 (1.10–2.48)	1.25 (0.65–2.37)
Black, non-Hispanic	1.66 (1.16–2.36)	2.35 (1.59–3.50)
Hispanic	1.25 (0.93–1.69)	1.70 (1.22–2.36)
Other/multiracial, non-Hispanic^c^	0.99 (0.76–1.28)	0.95 (0.63–1.42)
White, non-Hispanic	1 [Reference]	1 [Reference]
**Maternal age**, y		
≤24	1.13 (0.80–1.59)	1.32 (0.90–1.95)
25–29	1.47 (1.11–1.96)	1.24 (0.89–1.75)
30–34	1.14 (0.94–1.38)	1.10 (0.86–1.40)
≥35	1 [Reference]	1 [Reference]
**Caregiver education**		
High school graduate/GED or less	2.06 (1.52–2.80)	2.81 (2.00–3.95)
Some college or technical school	1.55 (1.24–1.94)	2.11 (1.57–2.84)
College degree or higher	1 [Reference]	1 [Reference]
**Household income^d^**		
<130% FPG	1.32 (0.99–1.77)	1.61 (1.06–2.43)
130% to <350% FPG	1.25 (1.03–1.52)	1.28 (0.92–1.78)
≥350% FPG	1 [Reference]	1 [Reference]
**Primary household language**		
English	1 [Reference]	1 [Reference]
Spanish	1.84 (1.14–2.99)	1.93 (1.17–3.18)
Other	0.87 (0.51–1.48)	1.14 (0.58–2.24)
**Household food situation during the past 12 months**		
Food sufficient: could always afford to eat good nutritious meals	1 [Reference]	1 [Reference]
Marginal food sufficiency: could always afford enough to eat but not always nutritious meals	1.09 (0.86–1.38)	1.73 (1.32–2.28)
Low food sufficiency: often or sometimes could not afford enough to eat	0.79 (0.44–1.39)	1.30 (0.71–2.38)
**Frequency of family eating meals together during the past week**		
0–3 days/week	1.43 (1.12–1.83)	1.40 (1.06–1.84)
4–6 days/week	1.28 (1.06–1.54)	1.21 (0.92–1.60)
Every day	1 [Reference]	1 [Reference]
**Metropolitan statistical area status**		
Metropolitan statistical area	1 [Reference]	1 [Reference]
Nonmetropolitan statistical area	1.23 (0.98–1.56)	1.39 (1.08–1.78)
**Government food assistance benefits for anyone in the household in the past 12 months^e^**		
Yes	1.17 (0.94–1.46)	1.37 (1.06–1.76)
No	1 [Reference]	1 [Reference]

Abbreviations: FPG, federal poverty guidelines; GED, General Educational Development certificate.

a Based on the following question: “During the past week, how many times did this child drink sugary drinks such as soda, fruit drinks, sports drinks, or sweet tea? Do not include 100% fruit juice.”

b All variables were included in one multinomial logistic regression model (unweighted n = 16,209 children without missing data). The reference outcome category was consuming SSBs 0 times/week. Findings were considered significant if 95% CIs did not include 1.

c Included American Indian/Alaska Native, Native Hawaiian and other Pacific Islander, and multiple race.

d This variable represents household income as a percentage of the federal threshold by family composition. This variable was multiply imputed according to National Survey of Children’s Health guidelines to account for missing values. We recategorized the cutoffs to reflect the cut point for participation in the Supplemental Nutrition Assistance Program (SNAP) and provide relatively equal sample sizes for each income group.

e This variable reflects participation in SNAP or Special Supplemental Nutrition Program for Women, Infants, and Children (WIC) or free or reduced cost breakfast or lunch benefits in the previous 12 months.

Higher odds of high SSB intake were significantly associated with children being older (vs 1 year of age), non-Hispanic Black or Hispanic (vs non-Hispanic White), highest caregiver education of high school graduate/GED or less or some college or technical school (vs college degree or higher), household income of less than 130% (vs ≥350%) of FPG, primary household language of Spanish (vs English), marginal food sufficiency in the household (vs food sufficient), 0 to 3 family meals per week (vs every day), nonmetropolitan statistical area status (vs metropolitan statistical area status), and anyone in the household received government food assistance benefits in the past 12 months (vs no government food assistance benefits) (Table 2). Compared with non-Hispanic White children, non-Hispanic Black children were more likely to have high SSB intake (aOR, 2.35; 95% CI, 1.59–3.50). Children who lived in households with the highest caregiver education level of less than or equivalent to high school graduate were also more likely to have high SSB intake compared with those with who lived in households with highest caregiver education level of college or above (aOR, 2.81; 95% CI, 2.00–3.95).

## Discussion

This study investigated factors associated with SSB intake among US children aged 1 to 5 years of age by using recent, nationally representative data. SSBs are leading sources of added sugars and have been associated with adverse health outcomes (4–7). The recommended daily caloric intake for boys and girls aged 1 to 5 years ranges from 1,000 kcal/d to 2,000 kcal/d, and it is recommended that children aged 2 to 5 years limit intake of added sugars to less than 10% of total daily calories (1). Drinking 1 can or bottle (12 oz or 355 ml) of soda could provide about 147 calories from added sugars per day (18). Consuming 147 extra kcal/d from these added sugars alone could exceed the recommended limit of all added sugars for some children. Any consumption of SSBs exceeds the recommended limit of added sugars for children aged less than 2 years. Among children aged 1 to 5 years, we found that 36% consumed SSBs 1 to 3 times/week and 21% consumed SSBs 4 or more times/week. After adjustment, several factors were significantly associated with both moderate (1–3 times/week) and high (≥4 times/week) intakes of SSBs. Children from households with a household caregiver education level of high school graduate/GED or less had significantly higher odds of moderate and high SSB intake than did children from households with a household caregiver education level of a college degree or higher. Even after adjusting for multiple covariates, we found that among children aged 1 to 5 years, those who were older, were non-Hispanic Black, had lower household caregiver education level, had lower household income, spoke Spanish as the primary household language, and reported fewer family meals in the past week were more likely to have moderate or high SSB intake in 2021.

These findings are similar to previous studies among young children. The Feeding Infants and Toddlers Study 2016 found that a significantly larger proportion of children aged 2 to 3 years consumed SSBs than did 1-year-olds on the day of the survey (19). Among children aged 1 to 5 years, National Health and Nutrition Examination Survey (NHANES) data for 2011 through 2014 also showed a higher proportion of older children consuming SSBs on a given day, with the highest percentage among those aged 4 to 5 years, followed by those aged 2 to 3 years, and then those aged 1 year (20). Our findings are in line with studies in youth and adult populations (5,8,21,22) which found that being non-Hispanic Black, having lower socioeconomic status, having lower caregiver education, or living in nonmetropolitan areas were associated with higher SSB intake.

### Social ecological model

Identifying interventions to promote and support healthy beverage behaviors for young children could be an important step to help young children have optimal growth and development. The social ecological model (SEM) has been used for health promotion programs for decades to focus on factors that influence health at the individual, interpersonal, organizational, community, and policy levels (23). In recent years the SEM has been used as a framework to examine factors and interventions that may affect behaviors related to childhood obesity at each level (23). The factors we found to be associated with SSB intake could be considered within the SEM framework. Settings such as in pediatric health care facilities, early child education (ECE) centers, and in the home might be potential sites for focusing public health programs or educational campaigns.

### Individual and interpersonal levels

Efforts to reduce SSB intake and encourage healthy beverage choices among adolescents exist (24,25). However, fewer data are available on effective programs for children aged 1 to 5 years. A systematic review found that successful interventions reducing intake of SSBs among young children used 3 or more strategies to target SSB intake (25). These strategies included in-person, individual, and group education for parents and children, passive education including posters and other resources, leveraging technology, training childcare providers, and changing physical access to SSBs (25). Further research could help to confirm these findings and test the effectiveness of strategies to reduce SSB intake in varying populations, settings, and circumstances.

Another important setting to reduce intake of SSBs among young children is in the home (10). We found that the frequency of family meals was significantly associated with SSB intake. Previous studies have shown relationships between SSB intake and parental modeling and feeding practices, as child SSB intake is highly related to parental SSB intake (10). Educational and communication campaigns could focus on reducing consumption of SSBs in the home. Strategies to reach families could consider the impact of using stories from voices within various communities related to SSB consumption, media and social media influencers, and tool kits containing educational resources for organizations to use with families (26). SSB messaging in other key settings, such as pediatric health care and childcare providers, could also reinforce positive parental modeling in the home.

### Organizational and institutional levels

Our findings support an early start to counseling families on healthy beverage options for young children. The Bright Futures initiatives from the American Academy of Pediatrics provide tools for preventive screenings and health supervision visits, including recommendations to counsel patients and caregivers about added sugars and beverages high in sugar (27). Adding SSB intake screening questions to electronic health records (EHRs) could help initiate behavior change counseling during routine well-child visits. Research has shown that implementation is feasible and may increase frontline health care workers’ knowledge of pediatric beverage recommendations to better counsel families (28). Additionally, EHR data on SSB intake could be used as a surveillance tool to identify the prevalence and associations with other health conditions and factors (29).

Federal food assistance programs, such as SNAP, Supplemental Nutrition Assistance Program Education (SNAP-Ed), or WIC, or programs aimed at improving nutrition and providing nutrition standards for childcare settings like the Child and Adult Care Food Program (CACFP) may be an important resource for ECE programs to promote healthy beverage choices for young children (30–32). Young children who receive care from ECE programs such as centers, family childcare homes, Head Start, and pre-K programs can benefit from policies and practices that prohibit SSBs being served to children in care (33). In 2022, 34 states had regulations that required all licensed ECE centers to follow CACFP meal patterns, regardless of CACFP participation (33). The CACFP requirements related to avoiding sugary drinks and following nutrition standards can serve as an important avenue for improving young child nutrition overall and reducing SSB intake (32,34).

### Community and system or policies levels

Policies and programs that increase access to healthy options for families may be effective for reducing SSB intake among young children (23). Additionally, factors found to be associated with SSB intake, including geographic location, could be important for tailoring a program to a community’s needs. For instance, SSB intake was found to vary by metropolitan statistical area status, and interventions could consider what associated factors may be important for an urban or a rural environment. In addition, factors such as household language could be considered in conjunction with other intervention methods, such as creating nutrition labels in multiple languages, to ensure accessibility for varying populations (23). The environment in which a family resides may affect SSB intake among young children and can be considered when deciding which factors may be most effective for creating effective community level programs to support nutrition among young children (23).

### Strengths and limitations

This study has several strengths. The data are a unique contribution, providing timely nationally representative data, with an oversampling of children aged 1 to 5 years. Most previous studies on young children’s SSB intake relied on data before 2015. Data from this study were collected from June 2021 to January 2022, which also provides information during the COVID-19 pandemic (14). The larger sample size of children aged 1 to 5 years and a wide range of covariates allowed us to explore associations that have not been examined in the past (8,13,14). There are also limitations. First, data are cross-sectional, and causality cannot be inferred. Second, data were reported by a caregiver, thus there could be recall or social desirability bias and the caregiver might not know everything a child consumed. Third, SSB intake was assessed with only 1 question and might underestimate SSB intake. Based on a previous study conducted in adult populations, prevalence of daily SSB intake was significantly underestimated when a 1-question screener was used compared with a 4-question screener (35). Lastly, the quantity of SSBs consumed was not captured and frequency of intake was categorized by week in our study, whereas recommendations are for daily caloric intake, and therefore cannot be linked to meeting, or exceeding, recommendations (14).

### Conclusions

We found that 36% of children aged 1 to 5 years consumed SSBs 1 to 3 times in the previous week and 21% of children aged 1 to 5 years consumed them 4 or more times in the previous week in 2021. SSB intake among children aged 1 to 5 years was higher among those who were older, were non-Hispanic Black, were from households with lower caregiver education level and lower household income, had a primary household language of Spanish, had marginal food sufficiency in the household, had lower frequency of family meals, lived in a nonmetropolitan statistical area, and received government food assistance benefits in the past 12 months. Our findings provide a recent, nationally representative assessment of the association of select social, behavioral, and household characteristics with SSB intake in young children. Public health initiatives in various settings could consider these factors associated with SSB intake among young children in designing targeted interventions. Additional research could investigate associated factors further, such as by geographic regions or states, to inform public health practitioners and researchers in designing effective programs.

## References

[1] US Department of Agriculture; US Department of Health and Human Services. *Dietary Guidelines for Americans, 2020–2025.* 9th edition. Published December 2020. Accessed October 6, 2022. https://www.dietaryguidelines.gov/sites/default/files/2021-03/Dietary_Guidelines_for_Americans-2020-2025.pdf

[2] Stowe EW , Moore LV , Hamner HC , Park S , Gunn JP , Juan W , . Meeting the Healthy People 2030 added sugars target. *Am J Prev Med.* 2023;65(1):4–11. 10.1016/j.amepre.2023.02.004 36907748 PMC11290098

[3] Dietary Guidelines Advisory Committee. *Scientific Report of the 2020 Dietary Guidelines Advisory Committee. Advisory Report to the Secretary of Agriculture and the Secretary of Health and Human Services.* US Department of Agriculture, Agricultural Research Service; 2020. Accessed November 3, 2022. https://www.dietaryguidelines.gov/sites/default/files/2020-07/ScientificReport_of_the_2020DietaryGuidelinesAdvisoryCommittee_first-print.pdf

[4] Bailey RL , Fulgoni VL III , Cowan AE , Gaine PC . Sources of added sugars in young children, adolescents, and adults with low and high intakes of added sugars. *Nutrients.* 2018;10(1):102. 10.3390/nu10010102 29342109 PMC5793330

[5] Laniado N , Sanders AE , Godfrey EM , Salazar CR , Badner VM . Sugar-sweetened beverage consumption and caries experience: an examination of children and adults in the United States, National Health and Nutrition Examination Survey 2011–2014. *J Am Dent Assoc.* 2020;151(10):782–789. 10.1016/j.adaj.2020.06.018 32979957

[6] Rousham EK , Goudet S , Markey O , Griffiths P , Boxer B , Carroll C , . Unhealthy food and beverage consumption in children and risk of overweight and obesity: a systematic review and meta-analysis. *Adv Nutr.* 2022;13(5):1669–1696. 10.1093/advances/nmac032 35362512 PMC9526862

[7] Xie L , Atem F , Gelfand A , Delclos G , Messiah SE . Association between asthma and sugar-sweetened beverage consumption in the United States pediatric population. *J Asthma.* 2022;59(5):926–933. 10.1080/02770903.2021.1895210 33625285 PMC8846412

[8] Hamner HC , Dooyema CA , Blanck HM , Flores-Ayala R , Jones JR , Ghandour RM , . Fruit, vegetable, and sugar-sweetened beverage intake among young children, by state — United States, 2021. *MMWR Morb Mortal Wkly Rep.* 2023;72(7):165–170. 10.15585/mmwr.mm7207a1 36795611 PMC9949846

[9] Grimm GC , Harnack L , Story M . Factors associated with soft drink consumption in school-aged children. *J Am Diet Assoc.* 2004;104(8):1244–1249. 10.1016/j.jada.2004.05.206 15281041

[10] Mazarello Paes V , Hesketh K , O’Malley C , Moore H , Summerbell C , Griffin S , . Determinants of sugar-sweetened beverage consumption in young children: a systematic review. *Obes Rev.* 2015;16(11):903–913. 10.1111/obr.12310 26252417 PMC4737242

[11] Tasevska N , DeLia D , Lorts C , Yedidia M , Ohri-Vachaspati P . Determinants of sugar-sweetened beverage consumption among low-income children: are there differences by race/ethnicity, age, and sex? *J Acad Nutr Diet.* 2017;117(12):1900–1920. 10.1016/j.jand.2017.03.013 28495478 PMC5677590

[12] Park S , Blanck HM , Sherry B , Brener N , O’Toole T . Factors associated with sugar-sweetened beverage intake among United States high school students. *J Nutr.* 2012;142(2):306–312. 10.3945/jn.111.148536 22223568 PMC4532336

[13] Rosinger A , Herrick K , Gahche J , Park S . Sugar-sweetened beverage consumption among U.S. youth, 2011–2014. *NCHS Data Brief.* 2017;(271):1–8. 28135184

[14] US Census Bureau. About the National Survey of Children’s Health. Updated June 2023. Accessed October 6, 2022. https://www.census.gov/programs-surveys/nsch/about.html

[15] US Census Bureau. Questionnaires. Updated June 2023. Accessed October 6, 2022. https://www.census.gov/programs-surveys/nsch/technical-documentation/questionnaires.html

[16] US Census Bureau. Glossary. Updated June 2023. Accessed October 6, 2022. https://www.census.gov/programs-surveys/metro-micro/about/glossary.html

[17] US Census Bureau. National Survey of Children’s Health. 2021 National Survey of Children’s Health frequently asked questions. Published October 2022. Accessed October 6, 2022. https://www.census.gov/programs-surveys/nsch/data/datasets.html

[18] US Department of Agriculture. FoodData Central. Published April 2019. Accessed April 27, 2023. https://fdc.nal.usda.gov/fdc-app.html#/food-details/174852/nutrients

[19] Kay MC , Welker EB , Jacquier EF , Story MT . Beverage consumption patterns among infants and young children (0–47.9 Months): data from the Feeding Infants and Toddlers Study, 2016. *Nutrients.* 2018;10(7):825. 10.3390/nu10070825 29949886 PMC6073729

[20] Demmer E , Cifelli CJ , Houchins JA , Fulgoni VL III . Ethnic disparities of beverage consumption in infants and children 0–5 years of age; National Health and Nutrition Examination Survey 2011 to 2014. *Nutr J.* 2018;17(1):78. 10.1186/s12937-018-0388-0 30134909 PMC6106834

[21] Ogden CL , Kit BK , Carroll MD , Park S . Consumption of sugar drinks in the United States, 2005–2008. *NCHS Data Brief.* 2011;(71):1–8. 22617020

[22] Imoisili O , Park S , Lundeen EA , Pan L , O’Toole T , Siegel KR , . Sugar-sweetened beverage intake among adults, by residence in metropolitan and nonmetropolitan counties in 12 states and the District of Columbia, 2017. *Prev Chronic Dis.* 2020;17:E07. 10.5888/pcd17.190108 31971897 PMC6993784

[23] Jernigan J , Kettel Khan L , Dooyema C , Ottley P , Harris C , Dawkins-Lyn N , . Childhood Obesity Declines project: highlights of community strategies and policies. *Child Obes.* 2018;14(S1):S32–S39. 10.1089/chi.2018.0022 29565654 PMC5865627

[24] Yoshida Y , Simoes EJ . Sugar-sweetened beverage, obesity, and type 2 diabetes in children and adolescents: policies, taxation, and programs. *Curr Diab Rep.* 2018;18(6):31. 10.1007/s11892-018-1004-6 29671076 PMC6025796

[25] Vercammen KA , Frelier JM , Lowery CM , McGlone ME , Ebbeling CB , Bleich SN . A systematic review of strategies to reduce sugar-sweetened beverage consumption among 0-year to 5-year olds. *Obes Rev.* 2018;19(11):1504–1524. 10.1111/obr.12741 30019442

[26] Centers for Disease Control and Prevention. Communication strategies. Updated February 2023. Accessed May 5, 2023. https://www.cdc.gov/healthliteracy/researchevaluate/comm-strategies.html

[27] American Academy of Pediatrics. Bright Futures. Updated June 2022. Accessed February 21, 2023. https://www.aap.org/en/practice-management/bright-futures/

[28] Lewis KH , Skelton JA , Hsu FC , Ezouah P , Taveras EM , Block JP . Implementing a novel electronic health record approach to track child sugar-sweetened beverage consumption. *Prev Med Rep.* 2018;11:169–175. 10.1016/j.pmedr.2018.06.007 29988772 PMC6031146

[29] Lewis KH , Skelton J , Hsu F-C , Ezouah P , Taveras EM , Block JP . Use of electronic health record data to study the association of sugary drink consumption with child weight status. *Acad Pediatr.* 2020;20(6):767–775. 10.1016/j.acap.2019.11.002 31712182

[30] US Department of Agriculture. SNAP-Ed Connection. Accessed March 2023. https://snaped.fns.usda.gov/

[31] US Department of Agriculture, Food and Nutrition Service. Special Supplemental Nutrition Program for Women, Infants, and Children (WIC). Published 2016. Accessed March 2023. https://www.fns.usda.gov/wic

[32] US Department of Agriculture, Food and Nutrition Service. Child and Adult Care Food Program. Accessed March 2023. https://www.fns.usda.gov/cacfp

[33] Centers for Disease Control and Prevention. Early Care and Education State Indicator Report 2023. US Dept of Health and Human Services; 2023.

[34] Centers for Disease Control and Prevention. Advancing Farm to Early Care and Education (ECE). Published January 5, 2023. Accessed December 1, 2023. https://www.cdc.gov/obesity/strategies/farm-to-ece.html

[35] Lundeen EA , Park S , Dooyema C , Blanck HM . Total sugar-sweetened beverage intake among US adults was lower when measured using a 1-question versus 4-question screener. *Am J Health Promot.* 2018;32(6):1431–1437. 10.1177/0890117117736957 29121793 PMC6298428

